# Characterizing the Content Related to Oral Health Education on TikTok

**DOI:** 10.3390/ijerph182413260

**Published:** 2021-12-16

**Authors:** Laurie Fraticelli, Colette Smentek, Delphine Tardivo, Julien Masson, Céline Clément, Sylvain Roy, Claude Dussart, Denis Bourgeois, Florence Carrouel

**Affiliations:** 1 Health, Systemic, Process, UR 4129 Research Unit, University Claude Bernard Lyon 1, University of Lyon, 69008 Lyon, France; laurie.fraticelli@univ-lyon1.fr (L.F.); colettesmentek@yahoo.fr (C.S.); julien.masson@univ-lyon1.fr (J.M.); celine.clement@univ-lorraine.fr (C.C.); sroy.psychologue@wanadoo.fr (S.R.); claude.dussart@univ-lyon1.fr (C.D.); denis.bourgeois@univ-lyon1.fr (D.B.); 2 ADES UMR 7268, Aix-Marseille University, 13344 Marseille, France; delphine.tardivo@protonmail.com; 3 “Interpsy” Laboratory, University of Lorraine, EA 4432, 54015 Nancy, France

**Keywords:** oral health education, TikTok, young adult, prevention, social media

## Abstract

Neglecting oral hygiene in adolescents negatively affects dental caries and periodontal diseases, in addition to social and emotional well-being. Thus, the TikTok platform (ByteDance, Beijing, China)as a social media could be a powerful channel to provide health-related information and educational content. This study aims to assess the quality of the TikTok videos corresponding to #oralhealtheducation. Sixty-nine videos were identified. Three oral health professionals (OHP), three health education professionals (HEP), and ten of TikTok’s target audience watched and evaluated the videos from a qualitative questionnaire. OHP detected false or incorrect information in 11.6% (8/69) of the videos. At least two HEPs reported being unable to detect this type of content or whether the video met dental ethics standards in both the videos. Disagreement was observed among the professionals themselves. The evaluation indicated that TikTok’s target audience was satisfied with the products viewed with an average score of >2.5, unlike the professionals, whose average score was <2.5 on a scale of 0 to 5. Users are advised to think critically and to consider the content of TikTok oral health videos with caution. The involvement of health professionals in the writing and validation of the videos could be an added value to positively respond to the needs of the adolescents.

## 1. Introduction

Oral diseases represent a significant health burden for individuals and populations [1]. The advantage of these diseases is that they are preventable. Health promotion initiatives in children and youth have been shown to reduce the occurrence of these diseases. In the field of dentistry, health promotion remains limited. This may be due to the fact that the ethics and philosophy of dentistry are oriented toward a curative and individualized approach to oral disease, and that there is confusion about health promotion as a concept [2]. Although the need to shift the focus of health services from treatment to prevention has been recognized, little progress has been made in many settings. This is true for oral health, where evidence of effective preventive interventions has not been systematically harnessed in oral health services [3].

Oral health care education represents a public health care priority since maintaining a good level of oral hygiene is an essential part of overall health [4]. Oral diseases and particularly periodontal diseases are a risk factor of non-communicable diseases such as cardiovascular diseases, diabetes, rheumatoid arthritis, cancer, and chronic obstructive pulmonary disease [5]. In adolescents, neglect of oral hygiene is believed to be responsible for the high prevalence of dental caries, and periodontal disease have a high prevalence in addition to social and emotional well-being [6,7]. During childhood, parents and caregivers make the decisions since children are not sufficiently trained. In adolescence, young people begin to make and implement decisions on their own to achieve the best possible outcome [8]. However, poor eating habits and irregular dental checkups are associated with the development of dental disease [9]. Currently, no long-term evidence of the effectiveness of school-based educational interventions in preventing plaque buildup, dental caries, and gingivitis has been demonstrated [10].

The change in health behaviors has undergone a revolution with the growth of the digital environment [11]. Mobile health (mHealth) is defined by the World Health Organization’s (WHO) Global eHealth Observatory as public health and medical strategies assisted by mobile technology [12]. The use of social media (SM) is one of the most common activities of adolescents today [13]. For the WHO European Region, the use of video games appears to be a real opportunity in the context of NCD prevention although they may be associated with some health risks. Social media allow the dissemination of health information in different forms such as blogs, podcasts, tweets, Facebook pages, or posts and YouTube videos [14]. Social media companies are responsible for the well-being of users [15]. Social media is a source for adolescents to seek information about oral health [16].

Health-related audiovisual content can impact society. Regardless of the content, dramatic means are used in social network videos to capture the short attention span of the audience and to compensate for the almost endless supply of other videos. Different categories of videos may attract different users and thus have different characteristics [17]. The consequence is that popular science web videos are not always the most complex or profound ones [18]. Moreover, these videos can promote unscientific therapies and medications and may alter patients’ beliefs. Therefore, it is critical that consumers have an objective look at published information so that they can make effective health care decisions [19].

TikTok, formerly known as musical.ly was created in September 2016 in China [20]. This social media actually accounts for one of the newest successful Chinese social media applications in the world [15]. The government agencies are becoming increasingly active participants in social media [21,22,23,24,25]. Recently, the WHO introduced its own TikTok app to provide information about the coronavirus. Since TikTok is mainly used by adolescents, this social media could therefore be a powerful channel to provide health-related information and educational information to young people [26,27,28,29]. However, health information from TikTok videos often does not meet the necessary standards [30].

Although less popular than female reproductive health, and acne treatment, information on oral hygiene is present on the platform. Also, it is generally accepted that nutrition and diet should be included for a holistic health program since they are common risk factors for oral diseases [31]. The question is whether applications with oral hygiene educational content are consistent with evidence-based dentistry and provide a high-quality context for oral self-management in children and adolescents.

The aim of this study is to assess the quality of the content related to oral health education on TikTok platform, as well as to evaluate its possible impact on the young population.

## 2. Materials and Methods

### 2.1. Study Design

This study was designed as an observational retrospective study based on the content of TikTok videos. As a social media platform, TikTok provides access to short videos, from 3 to 60 s, shared by TikTok users. The videos are accessible from a mobile application or accessible online on a website without a necessary user account. The TikTok version was 20.2.5 (3 July 2021). This study did not require any regulatory approval. All respondents were informed of the process and purpose of the study. This research was performed in accordance with the STROBE guidelines (Supplementary S1).

### 2.2. Sample Videos

**Figure 1:**
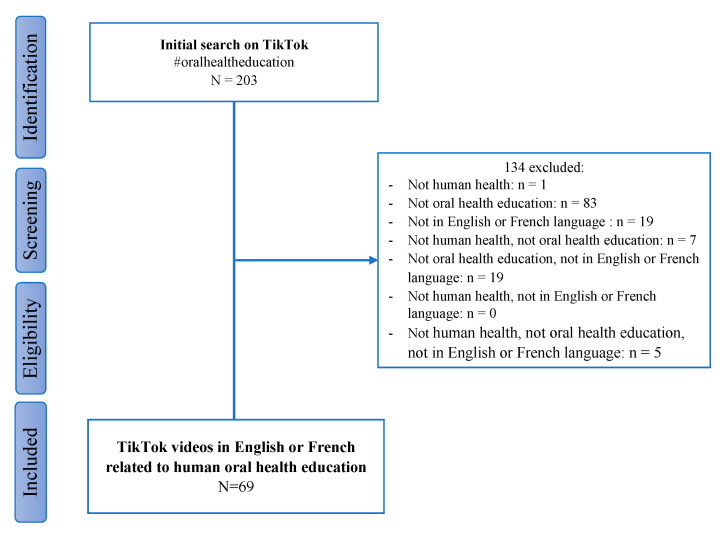
Chart flow of the TikTok videos corresponding to #oralhealtheducation.

A total of 203 TikTok videos were extracted with the hashtag #oralhealtheducation, which was associated with 6.8 million views on 28 September 2021 (Figure 1). Next, two university researchers (LF, FC) selected the videos. Videos were included if they (i) focused on human health, (ii) contained an educational or preventive message, advertising content, or commercial advice related to oral health, (iii) were in the French or English language. TikTok videos related to animal health, surgery, therapeutic or orthodontic treatment, piercing or tooth carving were excluded. After watching all the videos, 69 videos met the inclusion criteria and were selected for the evaluation study, representing 34%.

### 2.3. Survey Elaboration

Two academic researchers (LF, FC) specifically created an online questionnaire for the purpose of this study. This questionnaire was elaborated using the DISCERN instrument developed for judging the quality of written consumer health information on treatment choices [32], and from the PEMAT tool (patient education materials assessment tool) [33]. The aim of this survey was to describe and evaluate videos content with specific sections depending on the respondents’ profiles.

Section 1 focused on general data and contains two parts. The first one concerns the technical information of the TikTok video (5 items) and the second one concerns the overall description of the TikTok video (12 items). Section 2 analyzed the content of the video with one part on the objectives (7 items) and one part on the content of the video (12 items). Section 3 studied the subjective quality of the video (5 items).

The version was tested with a random selection of 10 videos, adjusted and finally approved by an oral health professional (DB) and a public health professional (DT). The final version of the questionnaire is available in Supplementary S2.

### 2.4. Evaluation of the Videos

After watching the 69 selected videos, two academic researchers (LF, FC) completed Section 1 from the online questionnaire. Then, all of the videos were evaluated by oral health professionals (OHP), health education professionals (HEP), and by young adults aged from 18 to 25 years old, representative of the TikTok’s target audience. The three OHP (DT, DB, CC) and three HEP (CS, JM, SR) completed Section 2 and Section 3 of the questionnaires, whereas the ten young adults answered only Section 3.

### 2.5. Statistical Analysis

The online questionnaire was constructed to be sure that all the raters provided an answer to each question; no missing data were allowed. For the categorical item, we provided the number of agreements (i.e., when all the raters quoted the same response) and the number of “yes” per agreement for categorical variables (yes/no). For the ordinal item, we provided the number of agreements on the five-item responses (1 totally disagree to 5 totally agree) and the number of raters who agree (score: 4) or strongly agree (score: 5).

To measure the inter- and intra-rater reliability between OHP and HEP, the Kappa of Fleiss (Kf) between categorical variables was used. A poor agreement corresponds to Kf < 0, a slight agreement to 0 <Kf < 0.2, a weak agreement to 0.2 < Kf < 0.4, an average agreement to 0.4 < Kf < 0.6, a good agreement to 0.6 < Kf < 0.8 and an excellent agreement to Kf > 0.8. The *p*-value indicates whether the calculated kappa is significantly different from zero and thus different from a random agreement; if the *p*-value is less than or equal to 5%, we conclude that the agreement between raters is significantly different from the value that would be obtained by chance.

For the ordinal variables, the intra-class coefficient (ICC) and its 95% confidence interval were calculated. An ICC close to 1 indicates high similarity between responses in the same group, while an ICC close to zero means that values in the same group are not similar.

Statistical analysis was performed using the package “irr” of the R Project for Statistical Computing (version 4.1.1. 2021-08-10).

## 3. Results

### 3.1. Technical Description of the Videos

The oldest video was posted 515 days ago, on 1 May 2020. We estimated that one video with the hashtag #oralhealtheducation was shared every two weeks. The median length of the included videos was 20 s [IQR 12;39], with 44.9% of videos (31/69) that mentioned at least one commercial brand. The median number of likes was seven [18;91]. Only 6 videos received more than 1000 likes (6/69, 8.7%), including 4 videos posted in the last 6 months.

#### 3.1.1. Video Setting and Staging

Among the included videos, 88.4% featured at least one person (61/69) and 11.6% did not feature anyone (8/69). Only one video featured more than five people. The main character presented himself as a health professional (40/61, 65.5%) due to his pseudonym in 24.6% of the included videos (15/61) or due to his professional outfit when at least one person is featured in 54.1% (33/61). The scene took place in a dental office in 15.9% of the videos (11/69) and 27.5% of the videos presented dummy material (mouth, gum, tooth or another object) (19/69).

Concerning the staging of videos, 73.9% had a musical background (51/69), 17.4% were entirely subtitled (12/69) and 84.0% were composed of key text-messages (58/69). The humor tone was used in less than 6% of the videos (4/69). The video setting and staging characteristics are described in Table 1.

#### 3.1.2. Oral Care Products Presented in the Videos

Oral care products presented or used in the videos are described in Table 2. Concerning the tooth cleaning, 50.0% of the videos included the toothbrushes as the main product described or used (35/69), and more particularly, the manual toothbrushes (26/35, 74.3%). The toothpaste and the mouthwash were proposed respectively in 27.1% (19/69) and 20.0% (14/69) of the videos. Concerning interdental cleaning, dental floss was the most used in the videos (16/69, 22.8%). Other products such as essential oils (1/69, 1.41%), tooth cleaner (1/69, 1.41%), spray (1/69, 1.41%), gel (1/69, 1.41%), chewing gum (1/69, 1.41%), whitening treatment (1/69, 1.41%) and night guard (1/69, 1.41%) were also described.

### 3.2. Intended Objectives by TikTok Videos Corresponding to #Oralhealtheducation

The analysis of the objectives of the video in relation to oral health education are presented in Table 3. Responses from OHP and HEP were analyzed in parallel. For each question, the responses of OHP were identical in at least 62.3% of the videos. The concordance of their answer ranged from slight agreement (Kf = 0.279) for the question C3 to important agreement (Kf = 0.757) for the question C2. The responses of HEP were identical in at least 36.2% of the analysed videos and the kappa of Fleiss ranged from low agreement (Kf = 0.0995) for the question C7 to average agreement (Kf = 0.535) for the question C2. The inter-rater agreement varied from weak for question C1 to good for question C2. We finally observed significant *p*-values when comparing the whole responses, regardeless of OHP and HEP groups.

**Figure 2:**
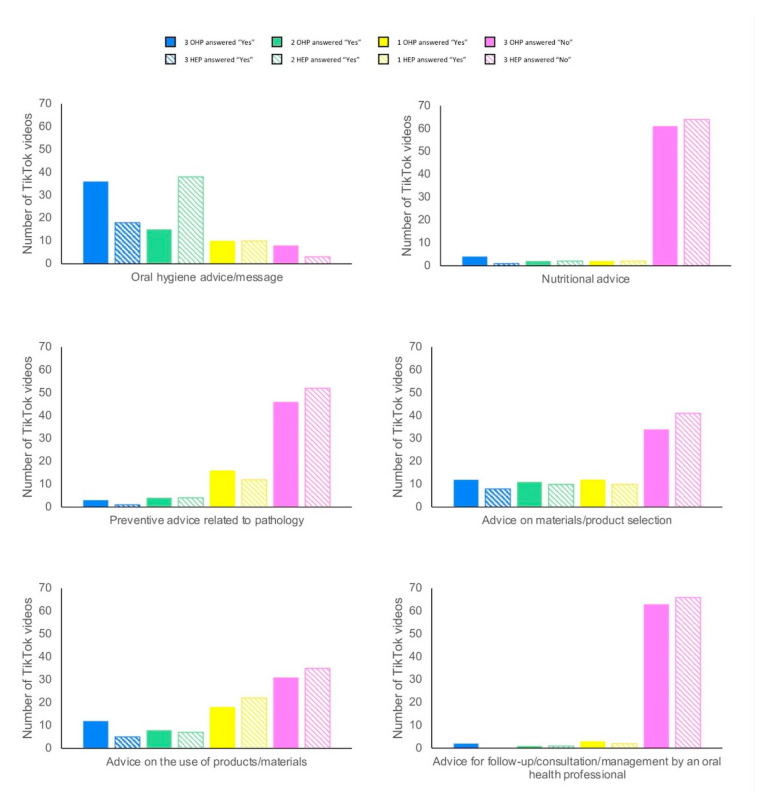
Targeted audience by TikTok videos corresponding to #oralhealtheducation.

The OHP and HEP agreed to concerning the three main objectives of the TikTok videos corresponding to #oralhealtheducation (Figure 2). The first goal of the studied videos was to provide oral hygiene advice or message (36/69, 52.2% of videos according to OHP; 21/69, 30.4% of videos according to HEP). The second one was to provide advice on materials/product selection (12/69, 17.4% of videos according to OHP; 8/69, 11.6% of videos according to HEP). And the third one was to provide advice on the use of products and/or materials (12/69, 17.4% of videos according to OHP; 5/69, 7.2% of videos according to HEP). The advice concerning the follow-up, the consultation, and the management was the objective of only two videos (2.9%) according to OHP and of no video according to HEP.

### 3.3. Targeted Audience by TikTok Videos Corresponding to #Oralhealtheducation

The target audience for the TikTok videos corresponding to #oralhealtheducation is summarized in Table 4. The OHP demonstrated an average agreement (Kf = 0.412) whereas the HEP had a slight agreement (Kf = 0.0995). The global inter-rater agreement was slight (Kf = 0.141).

The public targeted by Tiktok videos was clearly identified in only 13% of videos (9/69) for OHP and in 4% of videos (3/69) for HEP.

### 3.4. Scientific or Clinical Basis of the Message Delivered by TikTok Videos Corresponding to #Oralhealtheducation

Table 5 analyzes the message delivered by Tiktok videos corresponding to #oralhealtheducation. Responses from OHP and HEP were analyzed in parallel. The concordance of the OHP responses ranged from poor agreement (Kf = −0.0615) for the question D9 to good agreement (Kf = 0.713) for the question D12. The kappa of Fleiss for HEP ranged from poor agreement (Kf = −0.050) for the question D11 to weak agreement (Kf = 0.312) for the question D3. The inter-rater agreement varied from poor for question D10 to average for question D12.

According to OHP, the oral health subject was mastered in 28,9% (20/69) of videos and the vocabulary used was appropriated in 36.2% (25/69) of videos. They agreed that 42.0% (29/69) of videos complied with dental ethics or dental professionals and 28.9% (20/69) of videos applied official recommendations. They detected false or erroneous information in 11.6% (8/69) of videos. HEP evaluated that the oral health subject was mastered in 4.3% (3/69) of videos but at least one HEP answered “Do not know” in 84.1% (58/69) of videos. They judged that vocabulary used was appropriate in 8.7% (6/69) of videos but at least one HEP didn’t know in 68.1% (47/69) of cases. For all the videos, at least two of the three HEP declared to be unable to detect false or erroneous information or to know if the video respected dental ethics or dental professionals.

### 3.5. Subjective Evaluation of the Quality of TikTok Videos Corresponding to #Oralhealtheducation

**Figure 3:**
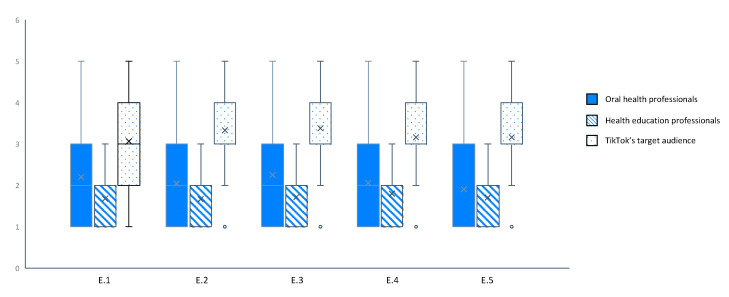
Subjective evaluation of TikTok videos corresponding to #oralhealtheducation. The Boxplot representing the quotation for question E.1 to E.5. E.1: Do you think the video is attractive?; E.2: Do you think the video is likely to increase awareness of the importance of oral health?; E.3: Do you think that video is likely to provide relevant information related to oral health?; E.4: Do you think the video is likely to change oral hygiene habits?; E.5: Overall score for the video.

The subjective evaluation of the quality was realized by OHP, HEP and TikTok’s target audience. The results are presented in Table 6. The number of agreements is low between OHP (<30.4%) and between HEP (<31.9%). Inter-rater agreement was low for all questions except for the attractiveness question where agreement was low. Despite a poor number of agreements among the TikTok’s target audience due to a larger sampling, we observed that the ICC were closed to 1 indicating a high similarity between responses. When the TikTok’s target audience was agreed, they were mostly agreed or totally agreed (4—agree/5—totally agree), as shown in Figure 3.

The average score given by TikTok’s target audience was always higher than OHP which was always higher than HEP. The average score given by the TikTok’s target audience was always higher than 2.5 whereas for the OHP end the EHP, the average score was always lower than 3. For the TikTok’s target audience, the videos were able to increase awareness of the importance of oral health and to provide relevant information related to oral health.

## 4. Discussion

Social media use among teens is nearly ubiquitous [34]. TikTok, the short video sharing social network, currently represents one of the most popular social media applications in the world with approximately 62% of all users being between the ages of 10 and 29 years [35]. Despite its success in terms of user numbers, studies to understand TikTok usage are scarce [20]. Especially since it is unlikely that all social media search results are applicable to TikTok. Each social media platform has a unique design, attracts different user groups [36], and causes a different potential for immersion or “addiction” [37] or misinformation.

The choice of the hashtag #oralhealtheducation allowed us to focus our research on videos that focused on oral health promotion and were targeted to the general population. The #oralhealth that reached a larger audience was not specific enough to the oral health promotion that was the focus of this article. It referred to numerous videos dealing with the curative aspect (treatment of cavities, periodontal diseases, orthodontics...) or the aesthetic aspect and was very often addressed to professionals and not to the general population.

Oral health education will not be a major target of the TikTok Health platform in 2021. The hashtag #oralhealtheducation corresponded to 203 TikTok videos and only 69 were in English or French and related to human oral health education. These videos received 141,720 “likes”, were commented 1358 times and shared 3295 times. Compared with other health-related topics, oral health education doesn’t appear to be of high interest for TikTokers and viewers. For example, the keyword “diabetes” referred to 199 TikTok videos which received 2.75 million “likes” and were commented on and shared thousands of times [30].

Our research is relevant to analyze oral health education-related videos on TikTok as well as their potential effect on behavior. TikTok users are often adolescents and therefore belong to a potentially vulnerable and influenceable group of individuals [30]. Moreover, oral health is one of the most unmet health care needs of adolescents. In addition to the usual problems of cavity management, dental referrals, and sports injury prevention, teenagers have specific oral health needs. The adolescence is associated with a high sugar and acid diet, the orthodontic treatments, the start of smoking and the oral piercings. Therefore, adolescents need a specific approach to get them interested in oral health issues [38].

In some cases, such as childhood caries, general population interventions (water fluoridation, etc.) have been shown to be more successful than population-specific and individual interventions [39]. TikTok videos could thus contribute to this reduction in inequalities. To be efficient, the oral health education strategy must be implemented early in life and thus must target a young audience, especially children and adolescents [40,41]. The first line of defense against the apparition of oral diseases such as caries and periodontal diseases has traditionally been patient education, with a special emphasis on optimal oral hygiene [42]. Guidelines for oral hygiene education are consensual. They are mainly: (i) toothbrush at least twice daily with a fluoride toothpaste, (ii) control consumption of sugar, (iii) control the consumption of tobacco, (iv) supplement with fluoride if concentrations in the primary water supply are insufficient, and (v) visit regularly the dental professional [43,44,45]. Recently, the use of interdental brushes has been highlighted to reduce the risk of interdental dysbiosis, inflammation and consequently the risk of interproximal caries and periodontal diseases [46,47,48]. Dietary guidelines to reduce food intake-snacking—are also of high relevance [49] since the oral health is associated with a healthy diet [31]. This is especially important since the eating habits acquired during the college years will impact the foundation of a lifelong lifestyle [50].

The TikTok videos were mainly focused on oral hygiene tips which is a one key element of the prevention of oral diseases [43,44,45]. The commercial brands do not seem to use TikTok as a direct way for advertising since there was only one sponsored video among the 69 selected videos. The commercial brands can indirectly use TikTok with “influencers” (i.e.,TikTok users with followers) to present their material or product, as observed in half of the content viewed (38/69).

Fifty percent of the videos included images of toothbrushes, with manual toothbrushes being overrepresented (74.3%). However, basic messages such as the indication of type of toothbrushes including the hardness of the filaments [51,52] and other technical criteria, are not addressed. Similarly, we noted the absence of advice on brushing technique, which should address the 45-degree angulation where the tooth and the gingiva meet, and the softness of brushing to prevent tooth and gingival trauma [53]. Thus, these videos do not provide consistent additional information about oral hygiene relative to adolescent challenges, and continue to support the concept that, without individualized education, people who usually use a manual toothbrush have difficulty achieving oral cleanliness [54,55,56,57,58]. The information provided on the indication of electric toothbrushes and their use is along the same direction, while it would be necessary to produce guidance to facilitate the fight against abrasiveness [59,60].

Among the oral hygiene components, the interdental thread is presented (30.4%). As previously described, there is a lack of criteria on the indications, choice and methods of use of the product. The floss is the interdental device the more present in the videos (16 of the 21 videos including interdental device). Cochrane Review on flossing underlined there is some evidence that flossing added to toothbrushing in adults may reduce gum disease and plaque compared to toothbrushing alone. But, overall, the evidence was low to very low- certainty [61]. Moreover, the correct use of dental floss is difficult [62]. On the other hand, the interdental brushes (21.3% of videos including interdental devices) would be more effective in reducing plaque, dysbiosis of the interdental microbiota, interdental inflammation and gingivitis [46,62]. Interdental brushes have the advantage of being easy to use and acceptable to the patient [62]. In addition, the adolescents are usually under orthodontic treatment. The most common negative effect of orthodontic treatment with fixed appliances is the development of incipient carious lesions around the brackets, periodontal damage by promoting gingivitis, gingival recession, and open gingival embrasures [63]. To prevent tooth and gum infections, people with brackets should consider using interdental brushes as part of their daily oral hygiene routine since they clean between teeth or around brackets, where a traditional toothbrush cannot reach.

Concerning tongue cleaning, the description of tongue scraper in the videos corresponding to hashtag #oralhealtheducation is interesting due to the fact that its efficiency was proven to fight against halitosis and gingivitis [64,65]. 

The use of chemical agents such as toothpaste and mouthwash was also presented in the videos (47.8%). Broadly speaking, there are two types of mouthwash: cosmetic and therapeutic. Cosmetic mouthwash may temporarily control bad breath and leave behind a pleasant taste, but have no chemical or biological application beyond their temporary benefit. Therapeutic mouthwash, by contrast, has active ingredients intended to help control or reduce conditions like bad breath, gingivitis, plaque, and tooth decay. This product contains active molecules that can help to fight against oral diseases such as caries or periodontal diseases but can help to fight against systemic diseases such as COVID-19 [66,67,68,69]. An update of the Cochrane review of fluoride mouthwashes for preventing dental caries in children and adolescents found that supervised regular use of fluoride mouthwash by children and adolescents is associated with a large reduction in caries increment in permanent teeth (the quality of evidence is moderate) [70]. The TikTok videos do not specify the targeted individuals or prescriptions (i.e., dose, frequency, time in mouth).

In terms of oral health education, nutrition and diet should be included in a global health program as they constitute common risk factors for non-communicable diseases [71,72]. Reviews of the literature have concluded that the use of nutrition-related mobile applications increases knowledge and improves behavior [73,74]. In our study, nutrition is mentioned in 0.06% of the videos analyzed. This lack of information concerning the diet should be considered since a healthy diet represents another key factor of oral health [31,75]. The choice of the #oralhealtheducation could in part explain the low number of video focusing on nutrition even if nutrition is a key factor of oral health [75].

From our analysis of the dental hygiene products contained in TikTok, it appears that even professionals have difficulty distinguishing whether the information contained in the videos comes from reliable sources. To stay safe from false or harmful content, creators on TikTok should clearly indicate where their information comes from, and users should remain vigilant [76]. Likewise, oral health providers, educational institutions, scientific societies, dental associations should be involved in creating quality information on TikTok and educate patients about misinformation to best support health literacy [76]. The WHO, in a broader health framework, has thus asked for the collaboration of social networks to fight against misinformation. It has recently subscribed to TikTok to provide quick and accurate information and reliable advice on public health [77].

Evaluation criteria are needed regarding the quality of the video that is uploaded by each video uploader about oral health education on TikTok, with the hope that viewers can receive useful information, good quality content. The videos analyzed were mainly made by a TikToker that was presented as a health professional (65.5%) due to his pseudonym or his professional outfit. Moreover, the scene took place in a dental office in 15.9% of the videos. Although TikTok has no means of monitoring to confirm that contributors are accredited health professionals, it was found that the number of health professionals using TikTok for health communication and awareness campaigns was increasing [28]. 

Even if the content of the TikTok videos related to oral health education is interesting, it is important to analyze how the message was delivered. The OHP evaluated that the subject was mastered in less than one third of the videos and the vocabulary appropriated in a little more than a third of the videos. The HEP evaluated that the subject and the vocabulary was correct in respectively 4.3% and 1.4% of the videos. This difference between the professionals demonstrated the difficulty to evaluate the TikTok videos contain. OHP did not agree concerning the fact that TikTok videos applied or not official recommendation (49.3% of agreement) and the fact that false or erroneous information was presented or not (46.4% of agreement). The evaluation of other TikTok videos in other health areas, such as “diabetes” or “acne”, has already shown serious shortcomings as well as the risk of spreading misinformation [30,78].

In addition, the subjective evaluation demonstrated that TikTok’s target audience and professionals have totally different demands and expectations in terms of content and staging. The TikTok’s target audience were satisfactory with the TikTok videos corresponding to #oralhealtheducation whereas the professionals were not. The TikTok’s target audience quoted the videos as (i) attractive, (ii) able to increase awareness of the importance of oral health, (iii) to provide relevant information related to oral health, and (iv) able to change oral hygiene habits. So, TikTok’s target audience that have no formation in the oral health domain cannot differentiate between reliable content and potentially biased content and should be more careful with the content of the videos related to oral health education.

Future audiovisual productions should be oriented to professional and domestic environments. In the oral healthcare sector, several fundamental trends are driving the adoption of advanced digital solutions to continue in the process of excellence. Advances in technology, prevailing patient preferences and dental school curriculums, as well as emerging treatment guidelines, are accelerating the adoption of future audiovisual productions with a simplified digital format. Access to quality continuing education, targeted to the specific needs of practitioners, is fully compatible with the principles that guide social media. They will also reduce inequalities in access to training for dentists. Social media must continue to be developed in the domestic environment with tools, such as TikTok, targeting teens and the nomad population. The framework is to empower, promote oral health and knowledge in the detection and self-management of the most common oral symptoms or diseases with a universal language, both of which are currently lacking. The content will have to evolve towards more rigor and scientific quality and evidence-based information, with full transparency regarding conflicts of interest.

There are several strengths to our analysis. This study is the first to analyze the TikTok videos corresponding to the hashtag #oralhealtheducation. Moreover, the point of view of three different groups of viewers (OHP, HEP, and young) was compared. However, this study also has limitations. First, the videos were only identified using #oralhealtheducation, and additional hashtags such as #toothbrushing, #mouthwash, #teethcleaning, among other related hashtags, often co-occurring with #oralhealtheducation, might yield subsets of videos with differing content, sentiment, and levels of incorrect content and misinformation. Secondly, the videos were included only if they were in French or in English, which probably excluded some interesting videos. Thirdly, due to the fact that the health-related audiovisual can impact the society, it could have been interesting to add a group of audiovisual communication professionals and some more specific questions. Fourthly, it is not possible to know if the TikToker is an oral health professional since only his pseudonym or outfit could give some indication but without real evidence. Fifthly, teenagers are not probably the only targeted audience. Although the videos are intended for teenagers, the video metrics (number of likes, comments, forwards, etc.) do not determine whether the audience figures are teenagers or other target audiences. Sixthly, unlike other content platforms, such as Youtube, that have their own campaigns, the TikTok application does permit the evaluation of the impact of the content. Finally, this study included only 10 young adults and the questionnaire had few questions regarding subjective evaluation. It might be interesting to conduct another study with a larger panel of users and targeted questions about their perception and knowledge of oral hygiene before and after viewing TikTok videos.

## 5. Conclusions

Our results indicate that the content of TikTok videos corresponding to the hashtag #oralhealtheducation is not always reliable, as has already been demonstrated in other studies of health-related videos. Moreover, our study showed the difference of opinion between the professionals themselves but also with the users. Thus, we recommend that users be careful and critical about the content of videos related to oral health. The involvement of health professionals in the writing and validation of the videos could be an added value to positively respond to the needs of adolescents.

## Figures and Tables

**Table 1:** Setting and staging characteristics of TikTok videos corresponding to #oralhealtheducation.

	n/N ^1^ (%)
**Concerning the main character:**	
Presented himself as a health professional	40/61 (65.6)
Pseudonym with “Dr” or “Doc” or “Doctor”	15/61 (24.6)
Dressed in a professional outfit	33/61 (54.1)
**Concerning the equipment, the demonstration was made:**	
By one character on himself	4/20 (20.0)
By one character on another person	3/20 (15)
**Staging of the video:**	
Musical background	51/69 (73.9)
Composed with key text-messages	58/69 (84.0)
Contained lyrics	38/69 (55.1)
Entirely subtitled	12/69 (17.4)
Humorous tone	4/69 (5.8)
Academic tone	5/69 (7.2)

^1^ n, number of videos; N, total number of videos.

**Table 2:** Oral care products used in the TikTok videos corresponding to #oralhealtheducation.

	n/N ^1^ (%)
Toothbrush:	35/69 (50.0)
Manual toothbrush	26/35 (74.3)
Electric toothbrush	9/35 (25.7)
Toothpaste	19/69 (27.1)
Mouthwash	14/69 (20.0)
Dental floss	16/69 (22.8)
Interdental brush	5/69 (7.1)
Tongue scraper	3/69 (4.3)
Other (water floss, essential oils, whitener, nocturnal denture)	8/69 (11.6)

^1^ n, number of videos; N, total number of videos.

**Table 3:** Intended objectives of the Tik Tok videos corresponding to #oralhealtheducation.

	Oral Health Professionals			Health Education Professionals			
Does the Video Provide (N = 69) ^1^:	Number of Agreements n (%) ^2^	Number of Yes per Agreement n (%) ^3^	Kappa of Fleiss (Kf)	Number of Agreements n (%) ^2^	Number of Yes per Agreement n (%) ^3^	Kappa of Fleiss (Kf)	Inter-Rater Agreement K (f)
C.1. oral hygiene advice/message?	44 (63.8)	36/44 (81.8)	0.407 *p* < 0.0001	25 (36.2)	21/25 (72.0)	0.074 *p* = 0.2830	0.218 *p* < 0.0001
C.2. nutritional advice?	65 (94.2)	4/65 (6.2)	0.757 *p* < 0.0001	65 (94.2)	1/65 (1.5)	0.535 *p* < 0.0001	0.643 *p* < 0.0001
C.3. preventive advice related to pathology?	49 (71.0)	3/49 (6.1)	0.279 *p* < 0.0001	52 (73.4)	1/52 (7.7)	0.199 *p* = 0.0042	0.264 *p* < 0.0001
C.4. advice on materials/product selection?	46 (66.7)	12/46 (26.1)	0.504 *p* < 0.0001	49 (71.0)	8/49 (16.3)	0.499 *p* < 0.0001	0.466 *p* < 0.0001
C.5. advice on the use of products/materials?	43 (62.3)	12/43 (27.9)	0.439 *p* < 0.0001	36 (52.2)	5/36 (13.9)	0.201 *p* = 0.0038	0.284 *p* < 0.0001
C.6. advice for follow-up/consultation/management by an oral health professional?	65 (94.2)	2/65 (3.1)	0.616 *p* < 0.0001	66 (95.6)	0/66 (0)	0.235 *p* = 0.0007	0.405 *p* < 0.0001

^1^ N, the total number of TikTok videos analyzed was 69. ^2^ n, number of videos for which all the raters quoted the same response; %, number of videos for which all the raters quoted the same response/total number of videos analyzed. ^3^ n, number of videos for which all raters answered “yes”; %, number of videos for which all raters answered “yes”/number of videos for which all the raters quoted the same response.

**Table 4:** Targeted audience by Tik Tok videos corresponding to #oralhealtheducation.

	Oral Health Professionals			Health Education Professionals			
Does the Video Provide (N = 69) ^1^:	Number of Agreements n (%) ^2^	Number of Yes per Agreement n (%) ^3^	Kappa of Fleiss (Kf)	Number of Agreements n (%) ^2^	Number of Yes per Agreement n (%) ^3^	Kappa of Fleiss (Kf)	Inter-Rater Agreement K (f)
C.7. Is the audience for the video clearly defined/identifiable?	42 (60.9)	9 (21.4)	0.412 *p* < 0.0001	23 (33.3)	3 (13.0)	0.099 *p* = 0.0766	0.141 *p* < 0.0001
All audience	47 (68.1)	1 (2.1)	0.151 *p* = 0.0083	48 (69.5)	0 (0)	0.133 *p* = 0.0563	0.111 *p* < 0.0001
Children	53 (76.8)	1 (1.8)	0.208 *p* = 0.0001	66 (95.6)	5 (7.6)	0.834 *p* < 0.0001	0.396 *p* < 0.0001
Adolescents	56 (81.1)	0 (0)	0.083 *p* = 0.1250	67 (97.1)	0 (0)	0.324 *p* < 0.0001	0.035 *p* = 01520
Women (including pregnant women)	60 (86.9)	2 (3.3)	0.403 *p* < 0.0001	68 (98.5)	2 (2.9)	0.870 *p* < 0.0001	0.581 *p* < 0.0001
Parents	57 (82.6)	0 (0)	0.030 *p* = 0.5820	68 (98.5)	0 (0)	−0.004 *p* = 0.9440	0.003 *p* = 0.899

^1^ N, the total number of TikTok videos analyzed was 69. ^2^ n, number of videos for which all the raters quoted the same response; %, number of videos for which all the raters quoted the same response/total number of videos analyzed. ^3^ n, number of videos for which all raters answered “yes”; %, number of videos for which all raters answered “yes”/number of videos for which all the raters quoted the same response.

**Table 5:** Scientific or clinical basis of the message delivered by TikTok videos corresponding to #oralhealtheducation.

	Oral Health Professionals			Health Education Professionals			
	Number of Agreements n (%) ^1^	Number of Yes per Agreement n (%) ^2^	Kappa of Fleiss (Kf)	Number of Agreements n (%) ^1^	Number of Yes per Agreement n (%) ^2^	Kappa of Fleiss (Kf)	Inter-Rater Agreement K (f)
D.1. The subject is mastered	32 (46.4)	20 (62.5)	0.282 *p* < 0.0001	9 (13.0)	3 (33.3)	0.177 *p* = 0.0004	0.132 *p* < 0.0001
D.2. The vocabulary is appropriate	38 (55.1)	25 (65.8)	0.379 *p* < 0.0001	11 (15.9)	6 (54.5)	0.132 *p* = 0.0079	0.25 *p* < 0.0001
D.3. The video describes the cause/etiology/mechanisms of occurrence of the problem (s)	51 (73.9)	7 (13.7)	0.489 *p* < 0.0001	31 (44.9)	9 (29.0)	0.312 *p* < 0.0001	0.385 *p* < 0.0001
D.4. The video cites any official source (s)	65 (94.2)	1 (1.5)	0.409 *p* < 0.0001	55 (79.7)	1 (1.8)	0.298 *p* < 0.0001	0.26 *p* < 0.0001
D.5. The video applies official recommendations	34 (49.3)	20 (58.8)	0.32 *p* < 0.0001	0 (0)	0 (0)	0.084 *p* = 0.1260	0.017 *p* = 0.4210
D.6. The expected benefits of the recommendations described	37 (53.6)	5 (13.5)	0.256 *p* = 0.0002	26 (37.7)	8 (30.8)	0.208 *p* = 0.0001	0.239 *p* < 0.0001
D.7. The consequences of not following the recommendations are described/explained	51 (73.9)	2 (3.9)	0.381 *p* < 0.0001	34 (49.3)	4 (11.8)	0.257 *p* < 0.0001	0.307 *p* < 0.0001
D.8. The video describes how following the recommendations affect quality of life	55 (79.7)	0 (0)	0.148 *p* = 0.033	27 (39.1)	0 (0)	−0.049 *p* = 0.3620	0.036 *p* < 0.0001
D.9. The video explains the limitations of the tips/materials presented	57 (82.6)	0 (0)	−0.0615 *p* = 0.376	52 (73.4)	0 (0)	0.028 *p* = 0.6010	0.073 *p* = 0.0051
D.10. The video gives false or erroneous information	32 (46.4)	8 (25.0)	0.231 *p* = 0.0009	0 (0)	0 (0)	−0.042 *p* = 0.5420	−0.047 *p* = 0.0395
D.11. The video complies with dental ethics or dental professionals	33 (47.8)	29 (87.8)	0.147 *p* = 0.0349	0 (0)	0 (0)	−0.050 *p* = 0.3600	−0.043 *p* = 0.0599
D.12. The video indicates when to consult a professional	65 (94.2)	3 (4.6)	0.713 *p* < 0.0001	50 (72.5)	2 (4.0)	0.310 *p* < 0.0001	0.443 *p* < 0.0001

^1^ n, number of videos for which all the raters quoted the same response; %, number of videos for which all the raters quoted the same response/total number of videos analyzed. ^2^ n, number of videos for which all raters answered “yes”; %, number of videos for which all raters answered “yes”/number of videos for which all the raters quoted the same response.

**Table 6:** Subjective evaluation of TikTok videos corresponding to #oralhealtheducation.

	Oral Health Professionals			Health Education Professionals				TikTok’s Target Audience		
	Number of Agreements n (%) ^1^	Agree (4) or Totally Agree (5) n (%) ^2^	ICC [95% CI]	Number of Agreements n (%) ^1^	Agree (4) or Totally Agree (5) n (%) ^2^	ICC [95% CI]	Inter-Rater Agreement ICC [95% CI]	Number of Agreements n (%) ^1^	Agree (4) or Totally Agree (5) n (%) ^2^	ICC [95% CI]
E.1. Do you think the video is attractive?	12 (17.4)	0 (0)	0.20 [0.06; 0.36]	15 (21.7)	0 (0)	0.03 [−0.09; 0.18]	0.18 [0.09; 0.28]	1 (1.5)	1 (100)	0.60 [0.52; 0.69]
E.2. Do you think the video is likely to increase awareness of the importance of oral health?	15 (21.8)	0 (0)	0.21 [0.06; 0.37]	22 (31.9)	0 (0)	0.11 [−0.03; 0.26]	0.22 [0.13; 0.33]	1 (1.5)	1 (100)	0.58 [0.49; 0.67]
E.3. Do you think that video is likely to provide relevant information related to oral health?	12 (17.4)	0 (0)	0.24 [0.09; 0.4]	21 (30.4)	0 (0)	0.12 [−0.02; 0.28]	0.21 [0.12; 0.32]	2 (2.9)	1 (50)	0.70 [0.62; 0.78]
E.4. Do you think the video is likely to change oral hygiene habits?	15 (21.8)	0 (0)	0.29 [0.14; 0.45]	13 (18.8)	0 (0)	0.08 [−0.06; 0.23]	0.23 [0.142; 0.346]	3 (4.3)	3 (100)	0.67 [0.59; 0.75]
E.5. Overall score for the video	21 (30.4)	0 (0)	0.37 [0.22; 0.51]	17 (24.6)	0 (0)	0.03 [−0.09; 0.19]	0.25 [0.15; 0.36]	3 (4.3)	3 (100)	0.65 [0.57; 0.74]

^1^ n, number of videos for which all the raters quoted the same response; %, number of videos for which all the raters quoted the same response/total number of videos analyzed. ^2^ n, number of videos for which all raters answered “Agree (4)” or “Totally Agree (5)”; %, number of videos for which all raters answered “Agree (4)” or “Totally agree (5)”/number of videos for which all the raters quoted the same response.

## Data Availability

The data presented in this study are available on request from the corresponding author.

## Supplementary Materials

The following are available online at https://www.mdpi.com/article/10.3390/ijerph182413260/s1, Supplementary S1: STROBE Statement—checklist of items that should be included in reports of observational studies, Supplementary S2: Questionnaire for the evaluation of TikTok videos in oral health education.
