# Supplementary material for: Characterizing the Content Related to Oral Health Education on TikTok
Source: Int J Environ Res Public Health. 2021 Dec 16;18(24):13260. doi: 10.3390/ijerph182413260 (PMC8701928; doi:10.3390/ijerph182413260)
Supplement: Supplementary file 1 [file ijerph-18-13260-s001.zip › ijerph-1495240-supplementary.pdf]

S1. STROBE Statement—checklist of items that should be included in reports of observational studies

	Item No	Recommendation	Page No
**Title and abstract**	1	(*a*) Indicate the study's design with a commonly used term in the title or the abstract	1
		(*b*) Provide in the abstract an informative and balanced summary of what was done and what was found	1
**Introduction**			
Background/rationale	2	Explain the scientific background and rationale for the investigation being reported	1-2
Objectives	3	State specific objectives, including any prespecified hypotheses	2
**Methods**			
Study design	4	Present key elements of study design early in the paper	2
Setting	5	Describe the setting, locations, and relevant dates, including periods of recruitment, exposure, follow-up, and data collection	2-3
Participants	6	(*a*) *Cohort study*—Give the eligibility criteria, and the sources and methods of selection of participants. Describe methods of follow-up<br>*Case-control study*—Give the eligibility criteria, and the sources and methods of case ascertainment and control selection. Give the rationale for the choice of cases and controls<br>*Cross-sectional study*—Give the eligibility criteria, and the sources and methods of selection of participants	3
		(*b*) *Cohort study*—For matched studies, give matching criteria and number of exposed and unexposed<br>*Case-control study*—For matched studies, give matching criteria and the number of controls per case	NA
Variables	7	Clearly define all outcomes, exposures, predictors, potential confounders, and effect modifiers. Give diagnostic criteria, if applicable	3
Data sources/ measurement	8*	For each variable of interest, give sources of data and details of methods of assessment (measurement). Describe comparability of assessment methods if there is more than one group	*3-4*
Bias	9	Describe any efforts to address potential sources of bias	*3-4*
Study size	10	Explain how the study size was arrived at	3
Quantitative variables	11	Explain how quantitative variables were handled in the analyses. If applicable, describe which groupings were chosen and why	3-4
Statistical methods	12	(*a*) Describe all statistical methods, including those used to control for confounding	3-4
		(*b*) Describe any methods used to examine subgroups and interactions	3-4
		(*c*) Explain how missing data were addressed	
		(*d*) *Cohort study*—If applicable, explain how loss to follow-up was addressed<br>*Case-control study*—If applicable, explain how matching of cases and controls was addressed<br>*Cross-sectional study*—If applicable, describe analytical methods taking account of sampling strategy	NA
		(*e*) Describe any sensitivity analyses	NA

**Results**			
Participants	13*	(a) Report numbers of individuals at each stage of study—eg numbers potentially eligible, examined for eligibility, confirmed eligible, included in the study, completing follow-up, and analysed	4
		(b) Give reasons for non-participation at each stage	4
		(c) Consider use of a flow diagram	3
Descriptive data	14*	(a) Give characteristics of study participants (eg demographic, clinical, social) and information on exposures and potential confounders	4
		(b) Indicate number of participants with missing data for each variable of interest	3
		(c) *Cohort study*—Summarise follow-up time (eg, average and total amount)	NA
Outcome data	15*	*Cohort study*—Report numbers of outcome events or summary measures over time	*NA*
		Case-control study—Report numbers in each exposure category, or summary measures of exposure	*NA*
		Cross-sectional study—Report numbers of outcome events or summary measures	*NA*
Main results	16	(*a*) Give unadjusted estimates and, if applicable, confounder-adjusted estimates and their precision (eg, 95% confidence interval). Make clear which confounders were adjusted for and why they were included	5-10
		(*b*) Report category boundaries when continuous variables were categorized	9-10
		(*c*) If relevant, consider translating estimates of relative risk into absolute risk for a meaningful time period	NA
Other analyses	17	Report other analyses done—eg analyses of subgroups and interactions, and sensitivity analyses	
**Discussion**			
Key results	18	Summarise key results with reference to study objectives	10-11
Limitations	19	Discuss limitations of the study, taking into account sources of potential bias or imprecision. Discuss both direction and magnitude of any potential bias	11-12
Interpretation	20	Give a cautious overall interpretation of results considering objectives, limitations, multiplicity of analyses, results from similar studies, and other relevant evidence	12-13
Generalisability	21	Discuss the generalisability (external validity) of the study results	12-13
**Other information**			
Funding	22	Give the source of funding and the role of the funders for the present study and, if applicable, for the original study on which the present article is based	NA

*Give information separately for cases and controls in case-control studies and, if applicable, for exposed and unexposed groups in cohort and cross-sectional studies.

Note: An Explanation and Elaboration article discusses each checklist item and gives methodological background and published examples of transparent reporting. The STROBE checklist is best used in conjunction with this article (freely available on the Web sites of PLoS Medicine at http://www.plosmedicine.org/, Annals of Internal Medicine at http://www.annals.org/, and Epidemiology at http://www.epidem.com/). Information on the STROBE Initiative is available at www.strobe-statement.org.

S2. Questionnaire for the evaluation of TikTok videos in oral health education

SECTION 1 - General data

A- Technical informations concerning the TikTok video

A.1. What is the publication date of the video ? jj/mm/aaaa

A.2. How many *likes* are associated with the video ? _ _ _ , _ _ _ , _ _ _ , _ _ _

A.3. How many comments are associated with the video ? _ _ _ , _ _ _ , _ _ _ , _ _ _

A.4. How many times has the video been forwarded ? _ _ _ , _ _ _ , _ _ _ , _ _ _

A.5. How long is the video ? _ _ seconds

B- Overall description of the TikTok video

B.1. Is the video sponsored? ☐ Yes ☐ No ☐ Don't know

B.2. Does the video quote brands? ☐ Yes ☐ No ☐ Don't know

B.3. Does the video feature people? ☐ Yes ☐ No ☐ Don't know

If yes:

B3a. How many people are presents? _ _ persons

B3b. The main character presents himself as a health professional ☐ Yes ☐ No

B3c. The main character wears a white coat/uniform ☐ Yes ☐ No

B.4. Is the video filmed in a dental office? ☐ Yes ☐ No ☐ Don't know

B.5. Is a demonstration made in the video? ☐ Yes ☐ No ☐ Don't know

If yes:

B5a. Is a fictitious mouth/gum/teeth used for the demonstration?

☐ Yes ☐ No ☐ Don't know

B5b. One person/patient is used for the demonstration?

☐ Yes ☐ No ☐ Don't know

B5c. Does the main character demonstrate on himself?

☐ Yes ☐ No ☐ Don't know

B.6. Is there any material in the video?

☐ electric toothbrush ☐ manual toothbrush ☐ dental floss
☐ interdental brushes ☐ toothpick ☐ tongue scraper
☐ mouthwash ☐ toothpaste ☐ Other: ____

B.7. Is the video in music? ☐ Yes ☐ No ☐ Don't know

B.8. Is the video fully transcribed? ☐ Yes ☐ No ☐ Don't know

B.9. Does the video show the key messages in writing? ☐ Yes ☐ No ☐ Don't know

B.10. Is a person speaking in the video? ☐ Yes ☐ No ☐ Don't know

B.11. Is the video humorous? ☐ Yes ☐ No ☐ Don't know

B.12. Is the video academic? ☐ Yes ☐ No ☐ Don't know

SECTION 2 - Content of the video

C- Objectives of the video

Does the video provide

C.1. oral hygiene advice/message? ☐ Yes ☐ No ☐ Don't know

C.2. nutritional advice? ☐ Yes ☐ No ☐ Don't know

C.3. preventive advice related to pathology? ☐ Yes ☐ No ☐ Don't know

C.4. advice on materials/product selection? ☐ Yes ☐ No ☐ Don't know

C.5. advice on the use of products/materials? ☐ Yes ☐ No ☐ Don't know

C.6. advice for follow-up/consultation/management by an oral health professional?
☐ Yes ☐ No ☐ Don't know

C.7. Is the audience for the video clearly defined/identifiable? ☐ Yes ☐ No ☐ Don't know

If yes:

☐ All audiences ☐ Children ☐ Adolescents ☐ Pregnant women
☐ Smokers ☐ Other: ________

D- Content of the video message

D.1. Is the subject mastered? ☐ Yes ☐ No ☐ Don’t know

D.2. Is the vocabulary appropriate? ☐ Yes ☐ No ☐ Don’t know

D.3. Does the video describe the cause / etiology / mechanisms of occurrence of the problem(s)? ☐ Yes ☐ No ☐ Don’t know

D.4. Does the video cite any official source(s)? ☐ Yes ☐ No ☐ Don’t know

D.5. Does the video apply official recommendations? ☐ Yes ☐ No ☐ Don’t know

D.6. Are the expected benefits of the recommendations described? ☐ Yes ☐ No ☐ Don’t know

D.7. Are the consequences of not following the recommendations described / explained? ☐ Yes ☐ No ☐ Don’t know

D.8. Does the video describe how following the recommendations affect quality of life? ☐ Yes ☐ No ☐ Don’t know

D.9. Does the video explain the limitations of the tips/materials presented? ☐ Yes ☐ No ☐ Don’t know

D.10. Does the video give false or erroneous information? ☐ Yes ☐ No ☐ Don’t know

D.11. Does the video comply with dental ethics or dental professionals? ☐ Yes ☐ No ☐ Don’t know

D.12. Does the video indicate when to consult a professional? ☐ Yes ☐ No ☐ Don’t know

SECTION 3 - Subjective quality of the application

E- Personal appreciation of the video

E.1. Do you think the video is attractive?

Totally disagree				Totally agree
1	2	3	4	5
○	○	○	○	○

E.2. Do you think the video is likely to increase awareness of the importance of oral health?

Totally disagree				Totally agree
1	2	3	4	5
○	○	○	○	○

E.3. Do you think that video is likely to provide relevant information related to oral health?

Totally disagree				Totally agree
1	2	3	4	5
○	○	○	○	○

E.4. Do you think the video is likely to change oral hygiene habits?

Totally disagree				Totally agree
1	2	3	4	5
○	○	○	○	○

E.5. Overall score for the video:

☐ ☆ ☐ ☆☆ ☐ ☆☆☆ ☐ ☆☆☆☆ ☐ ☆☆☆☆☆
